# A biometric and ecologic comparison between *Artemia *from Mexico and Chile

**DOI:** 10.1186/1746-1448-2-13

**Published:** 2006-11-24

**Authors:** Thalía B Castro, Gonzalo Gajardo, Jorge M Castro, Germán M Castro

**Affiliations:** 1 Universidad Autónoma Metropolitana-Xochimilco. Departamento "El Hombre y su Ambiente Calzada del Hueso No.1100. Col. Villa Quietud 04960, D.F., Mexico; 2 Laboratory of Genetics & Aquaculture, Department of Basic Sciences. Universidad de los Lagos, Osorno, Chile

## Abstract

**Background:**

A preliminary biometric and ecologic database for the brine shrimp *Artemia *from Mexico and Chile is presented. The area abounds in small and seasonal ponds and large inland lakes, the latter mainly located in Mexico, although relatively large and isolated lakes are found in complex hydrological settings in pre-high plateau areas of Chile. This paper summarizes research efforts aimed at the localization, characterization, and evaluation of the aquaculture potential of *Artemia *populations in Mexico and Chile, which exhibit great habitat diversity (ponds, salterns, coastal lagoons, sea arms, coastal and inland lakes), contrasting weather conditions and different levels of isolation and human intervention.

**Results:**

This study covered locations between 29° north latitude (Baja California, Mexico) to 50° south latitude (Puerto Natales, Chile). Biological characteristics considered are species name, reproductive mode, cyst diameter, chorion thickness, and nauplius length, whereas ecological data include pond size, pH, salinity, temperature, and water ionic composition. *Artemia franciscana *is the only species found in Mexico, it exists together with *A. persimilis *in Chile, though separated geographically. Ecological differences in habitat exist between both regions but also within countries, a pattern particularly clear with regard to water composition. Surprisingly, a Mexican (Cuatro Ciénegas, *A. franciscana*) and a Chilean location (Torres del Paine, *A. persimilis*) share habitat characteristics, at least for the period when data were collected. The discriminat analysis for cyst diameter and nauplius length shows that *Artemia *from only one location match in cyst diameter with those from San Francisco Bay (SFB) (Point Lobos), and one (Marquez) is far apart from SFB and all the others. The Chilean locations (Pampilla, Cejar, Cahuil, Llamara, Yape) share cyst diameter, but tend to differ from SFB. The remaining Mexican locations (Juchitan, Ohuira, Yavaros) are well separated from all the others. With regard to nauplii length, populations tend to distribute in a relatively random manner, being Marquez the location differing the most in cyst diameter from SFB.

**Conclusion:**

This database will contribute to the knowledge of radiation centers and serves as a baseline for further biogeographic studies, population characterization, management, and monitoring of *Artemia *biodiversity. Likewise, the impact of colonization and translocations for aquaculture purposes can be better assessed with a baseline for reference. Mexico and Chile exemplify the need to increase and further integrate regional information to tackle fundamental problems underlying practical utilization of *Artemia*.

## Background

The brine shrimp *Artemia *is widely distributed in salt lakes, coastal lagoons, and solar saltworks in all continents, except Antarctica [1]. Since the initial record of 80 *Artemia *sites (Abonyi, 1915; Artom, 1922; Stella, 1933; Mathias, 1937; all cited in Persoone & Sorgeloos [2], the number has steadily increased, for example, Vanhaecke *et al*. [3] and Triantaphyllidis *et al*. [4], reported a total of 350 and 500 *Artemia *locations, respectively. The search for new *Artemia *populations, or locally adapted populations, is relevant to solve fundamental questions on population differentiation in stressful habitats, but also to counterbalance the decline of *Artemia *cysts, which are highly demanded for aquaculture [5]. Hence, the search for alternative *Artemia *resources has intensified in recent years, especially in large and productive inland lakes that are amenable to commercial exploitation.

This study gathers and compares biometric and reproductive data of *Artemia *populations from ecologically diverse ecosystems (ponds, salterns, salt ponds, coastal lagoons, sea arms, coastal and inland lakes) from Mexico and Chile, two countries with potential *Artemia *sources for aquaculture. Important morphological and geological changes, such as the joining of North and South America during the Tertiary and the rising of the occidental and oriental slopes of the Andes Mountains, have influenced the area in the past, greatly affecting the current distribution pattern of many aquatic species.

Two *Artemia *species are currently found in the Americas, *A. franciscana *(Kellog, 1906) and *A. persimilis *(Piccinelli & Prosdocimi, 1968). The former is widely distributed over the Americas, whilst the latter was thought to be restricted to Argentina [6]. However, the finding of *A. persimilis *in a very unusual site in the Chilean Patagonia [7] and later of *A. franciscana *in Argentina [8], changed the distribution scenario. Most populations in Chile and adjacent areas are recognized as *A. franciscana*, but exhibit varying degrees of genetic differentiation in relation to the commonly used *A. franciscana *type (San Francisco Bay, Salt Lake) [9-11]. This distribution pattern requires more systematic and careful data analysis of populations in radiation centers, since *A. persimilis *could be in the process of colonizing new habitats in Chile and other countries in South America, while *A. franciscana, *a very successful colonizer [1], has been translocated by aquaculture activities and is currently expanding its range in Europe and Asia. This database will be useful for the spatial and temporal monitoring of *Artemia *biodiversity, particularly considering the ability of *Artemia *to invade other environments, either by translocation for aquaculture purposes or through natural dispersal, as has been demonstrated in the western Mediterranean for *A. franciscana *[12,13].

## Results

### Artemia sites in Mexico

#### Habitat information

*Artemia *ranges from 32° and 14° north latitude, and between 117° (Northeast of Baja California) and 86° West (Isla Mujeres). Seventeen *Artemia *populations have been recorded so far in this country, 14 of them in coastal areas (4 in the Gulf of Mexico, 10 in the Pacific Coast) whereas the rest corresponds to inland habitats [14]. Table 1 describes the geographic location, size, and altitude of the *Artemia *sites studied so far in Mexico. The largest site is Guerrero Negro, in the Baja California Peninsula, with 33,000 ha, whereas San Jose, in the same Peninsula, is the smallest (0.5 ha) and comparable in size to San Crisanto in the Yucatan Peninsula. Texcoco, in the State of Mexico, is located at the highest altitude (2,250 m above sea level).

**Table 1:** Geographical location, altitude and size of *Artemia *sites in Mexico.

Site	State	Geographical coordinate	Altitude (m)	Size (ha)	References
La Salina	Baja California Norte	32°05'N 118°40'W	0	33	Del Castillo & Farfán (1997) [38]
San José	Baja California Norte	29°15'N 114°53'W	0		Correa (1991) [24]
Cuatro Ciénegas de Carranza	Coahuila	29°36'N 99°20'W	740	0.05	Castro *et al*. (1997) [19]
Guerrero Negro	Baja California Sur	27°30' – 28°N 113°45' – 114°25'W	0	33000	Data from Exportadora de Sal S.A. de C.V.
Isla del Carmen	Baja California Sur	26°0' N 111°40'W			Castro *et al *(1987) [23]
Salina tres Hermanos (Yavaros)	Sonora	26°40'N 109°35'W	0	40	Gallardo astro (1987) [26]. Abreu-Grobois (1987) [6]. Castro *et al*.(1996) [18] (1997) [21]. Correa (1991) [24].
Bahía de Ohuira (Ahome)	Sinaloa	25°36'N 109°02'W	10		Díaz (2000) [25]
Pichilingue	Baja California Sur	24°16'N 110°20'W			Castro *et al*. (1987) [23].
Bahía de Ceuta	Sinaloa	23°50'N 106°30'W	0	7140	Castro *et al*. (1997) [20]
El Barranco (Altamira)	Tamaulipas	22°36' N 97°52'W 22°35'N 97°54'W	0		Contreras (1987) [39]
Salinas de Hidalgo	San Luís Potosí	22°39'N 101°43'W	1777		Castro *et al*.(1989) [17] (2000) [14]
San Crisanto	Yucatán	21°15'N 89°10'W	0	0.05	Castro *et al*. (1987) [23]
Real de las Salinas	Campeche	20°02'N 90°14'W	10	63.16	Castro *et al*. (1998) [16]
Celestún	Yucatán	20°48' 90°15' 20°58'N 90°25'W	0	3000	Torrentera & Dodson (1995) [4]. Núñez(1999) [27]
Texcoco	Estado de México	19°32'N 99°00'W	2250	0.17	Castro(1993) [15], Gallardo astro (1987) [26], Enciso (1989) [40]
Las Coloradas	Oaxaca	15°33'N 95°33'W	0	aprox.50	Castro *et al*.(1995) [22]
Sistema lagunar de: Laguna del mar Muerto	Chiapas	15°58' – 16°30'N 93° – 94°30'W	0		Tena (1977) [28]

The main water component of biotopes studied so far is sodium chloride, with the exception of Cuatro Cienegas, State of Coahuila, in which sulphate predominates (Table 2).

**Table 2:** Main water parameters of Mexican *Artemia *sites.

Sites	Salinity (g/L)	Temperature (°C)	pH	Cl^- ^(Mg/l)	SO_4_^-2 ^(mg/l)	HC0_3_^- ^(mg/l)	CO_3_^- ^(mg/l)	Na^+ ^(mg/l)	K^+ ^(mg/l)	Ca^+2 ^(mg/l)	Mg^+2 ^(mg/l)	Date	Reference
La Salina	290	22	7									April-84	Del Castillo y Farfán (1997) [38]
San José	184	25	7.2									February-89	Correa(1991) [24]. Montaño & Buckle (1996) [34]
Cuatro Ciénegas de Carranza	100–300	27	7.7	28 930	61 440	9 150		24 790	3 130	0	13 280	01/04/1994	Castro *et al*. (2000) [14]
Guerrero Negro	120	18–22										22/02/1999	
Isla del Carmen													
Salina tres Hermanos	183	24	8.10									April-70	Ortega & Martínez(1987) [41]
Bahía de Ohuira		20–25	8.6										
Pichilingue	100–300	22	8.10										
Bahía de Ceuta	100–300	22	8.2	77 790	14 164	5 760						October-89	Castro (1980) [42].
El Barranco (Altamira)	165	21	8									01/02/1986	Contreras(1987) [39]
Salinas de Hidalgo	80	17	9.84	2 240	228	52	84	2 696	230			01/11/1985	
San Crisanto		26											Castro *et al*. (1987) [23]
Real de las Salinas	100–300	23.2	8.9	15 120	100	700	0	13 500	1 350	0	100	01/02/1998	Castro *et al*. (1998) [16]
Celestún	185	29.1	8.1		485		1348					1991–1992	Torrentera & Dodson (2004) [36]
Texcoco	40	15–16	7.6	1 438	26	610	48	15 590	20	322		June-90	Castro (1993) [15]
Las Coloradas	125	28	8.0									02/12/1992	Castro (1995) [22]
Sistema lagunar de: Laguna del mar Muerto	35	30										June-80	Tena (1977) [28]

#### Biological data

Of all populations recorded in this area, 13 have been studied with respect to reproduction (crosses with *A. franciscana*) and morphology, whilst fragmentary genetic information (allozyme and chromosome) is available. These populations combine the two usual modes of reproduction (encystment and ovoviviparity), depending on the local conditions [6,15-28].

Table 3 provides cysts, chorion thickness, and nauplii measurements. The El Marquez population in Oaxaca depicts the biggest cyst diameter and the thickest chorion, whilst samples from Bahia de Lobos and Celestun showed the smallest cysts and nauplii.

**Table 3:** Cyst diameter, chorion thickness and nauplius characteristics of *Artemia franciscana *populations in Mexico.

State	Sites	Full cyst Diameter (± SD)	Chorion thickness	Nauplius size (± SD)
Sonora	Tres Hermanos, Yavaros	229.1 ± 8.9	8.76	389.5 ± 15.3
Sinaloa	Sol de Fuego, Ohuira	266.3 ± 8.6	7.96	379.6 ± 19.4
Oaxaca	Juchitán	275.5 ± 13.0	8.26	450.3 ± 30.6
Campeche	Real de las Salinas	249.2 ± 8.1	9.98	465.3 ± 29.3
Coahuila	Cuatro Ciénegas	231.1 ± 4.3	9.11	472.4 ± 26.9
San Luís Potosí	Las Salinas de Hidalgo	292.3 ± 16.1	6.94	417.9 ± 23.3
Estado de Mexico	Texcoco	230.2 ± 4.5	8.93	422.6 ± 29.0
Yucatán	Celestún	211.9 ± 13.6	3.87	461.8 ± 13.5
Zacatecas	Zacatecas	230.2 ± 4.1	2.11	432.7 ± 15.7
Sonora	Bahía de Lobos	200.4 ± 5.5	3.07	391.9 ± 16.3
Quintana Roo	Puerto Morelos	238.3 ± 8.8	9.95	398.0 ± 8.2
Oaxaca	El Marquez	386.3 ± 6.6	10.78	419.0 ± 8.7
Yucatán	San Crisanto	247.4 ± 16.1	3.72	446.0 ± 12.4

The data are in micrometers (μm).

### Artemia sites in Chile

#### Habitat information

Table 4 displays geographical coordinates, size, and altitude of the *Artemia *sites arranged from north to south, with the *Salar *(saltflat) Surire (18° South latitude) and Laguna Amarga (50° South latitude) being at opposite ends. The former is at the highest altitude in the Americas (4,200 m above sea level) in the Atacama Desert, reputedly one of the most arid areas in the world [9]. The desert, located in the subtropical area of Chile and limited by the Pacific Ocean to the West and the Andes Mountains to the East, in the so-called pre-high plateau zone, has plenty of saltflats, which are complex hydrological units that very likely evolved from a lacustrine system through a combination of geological, morphological, hydrological, volcanic and climatic factors [19]. Such saltflats represent therefore interesting and unique *Artemia *sites due to their isolation, altitude, and extreme ecological conditions (5 to 40°C day-night variation in summer time) [9], that offer few chances for colonization and/or survival of foreign populations.

**Table 4:** Geographical location, altitude and size of the *Artemia *sites in Chile.

Sites	Federal entity	Locality	Geographical coordinates	Altitude (m)	Size (ha)	References
Salar de Surire	I Región	Salar de Surire	18°48'S 69°04'W	4200	144 km^2^	Zuñiga *et. al*. (1999)* [43]
Salar de Llamara	I Región	Llamara	21°18'S 69°37'W	850	10.000ha	*
Pozas de Playa Yape	I Región	Iquique	20°40'S 70°15'W	0	4–28 m^2^	*
Laguna Cejas (Salar de Atacama)	II Región	San Pedro de Atacama	23°02'S 68°13'W	2400		Gajardo & Beardmore (1993) [11]; Zúñiga *et al*. (1999) [43]
Poza Pampilla	IV Región	Coquimbo	29°58'S 71°22'W	0	9 m^2^	*
Poza Palo Colorado	IV Región	Los Vilos	31°58'S 71°25'W	0	10 m^2^	*
Salinas de Cahuil	VI Región	Pichilemu	34°48'S 72°10'W	100 mt	4.5	*
Laguna Amarga	XII Región	Puerto Natales	50°29'S 72°45'W	80	227,6 ha	Zúñiga *et al*. (1999) [43]

Laguna Amarga (50° south latitude) is closer to the sea and correspondingly exhibits relatively high concentrations of sodium chloride, whereas high-plateau inland lakes in northern Chile, such as Surire, are athalassohaline, with high content of sulphate (60.38 g L^-1^) and potassium (7.48 g L^-1^) (Table 5).

**Table 5:** Main water parameters of Chile *Artemia *sites.

Sites	Salinity (g/l)	Temperature (°C)	pH	Cl^- ^(Mg/L)	SO_4 _^-2 ^(mg/L)	HC0_3 _^- ^(mg/L)	CO_3 _^- ^(mg/L)	Na^+ ^(mg/L)	K^+ ^(mg/L)	Ca^+2 ^(mg/L)	Mg^+2 ^(mg/L)	Date	Reference
Salar de Surire	102	4,5	8,5	46 370	11 030	150	40	31 860	7 480	1 360	1 270	July-1994	Gajardo y Beardmore (1993) [11]
Salar de Llamara	167	28	7,5	43 600	17 440			35 810	2 130	450	560	March-1994	Zuñiga *et al*.(1999) [43]
Pozas de Playa Yape	56	30	8,5	54 920	8 490	40	0	30 470	1 230	1 190	3 650	July-1994	
Laguna Cejas (Salar de Atacama).	292	23	7,3	41 900	28 250	370	0,3	26 780	1 230	120	1 330	March-1994	
Poza Pampilla	45	17	10,1	55 460	7 800	360	50	29 770	1 180	1 090	4 460	August-1994	
Poza Palo Colorado	75	21,1	8,1	53 300	7 580	220	30	32 480	1 320	1 260	3 810	August-1994	
Salinas de Cahuil	115	37	7,8	54 780	7 690	380	20	31 380	1 100	1 210	3 520	February-1995	
Laguna Amarga	120	8,5	12	17 020	60 380	4 420	1 320	34 160	2 250	330	2 089	April-1995	

#### Biological data

The largest cysts diameter is found in Laguna Amarga (254.7 μm), whilst Poza Pampilla (220.5 μm) is the smallest. The longest and shortest nauplii are found in the Salar de Llamara (424 μm) and Poza Pampilla (395 μm), respectively (Table 6). Laboratory cross-breeding experiments show different degrees of reproductive output and varying ratios of offsprings in the form of cyst and nauplii, depending on whether crosses are within or between species [11,30-33].

**Table 6:** Cyst, chorion thickness and nauplius characteristics *of Artemia franciscana *and *Artemia persimilis *in Chile.

Sites	*Artemia *species	Full cyst Diameter (± SD)	Chorion thickness	Nauplius size
Laguna Cejar (Salar de Atacama)	*Artemia franciscana*	226.7 ± 9.1	6.15	451.7 ± 23.3
Salinas de Cahuil	*Artemia franciscana*	230.6 ± 8.9	6.7	399.3 ± 18.8
Pozas de Playa Yape	*Artemia franciscana*	241.0 ± 8.8	6.36	415.0 ± 26.1
Salar de Llamara	*Artemia franciscana*	232.0 ± 7.6	7.9	424.0 ± 19.7
Poza Pampilla	*Artemia franciscana*	220.5 ± 12.5	5.4	395.5 ± 22.4
Laguna Amarga	*Artemia persimilis*	254.7	-	413.3
Poza Palo Colorado	*Artemia franciscana*	236.8	-	417.5

The data are in micrometers (μm).

#### Data integration

**Figure 1:**
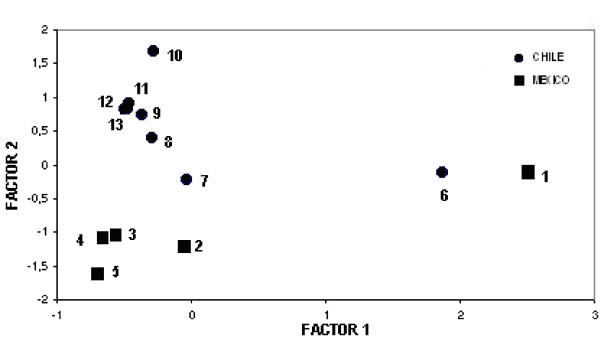
**Principal components analyses of water composition in different *Artemia *sites in Mexico-Chile**. Locations: 1 = Cuatro Cienegas, 2 = Bahía de Ceuta, 3 = Real de las Salinas, 4 = Texcoco, 5 = San Luis Potosí, 6 = Torre del Paine, 7 = Salar de Atacama, 8 = Salar de Llamara, 9 = Poza Pampilla, 10 = Salar de Surire, 11 = Poza Palo Colorado, 12 = Pozas de Playa Yape, 13 = Salinas de Cahuil.

**Figure 2:**
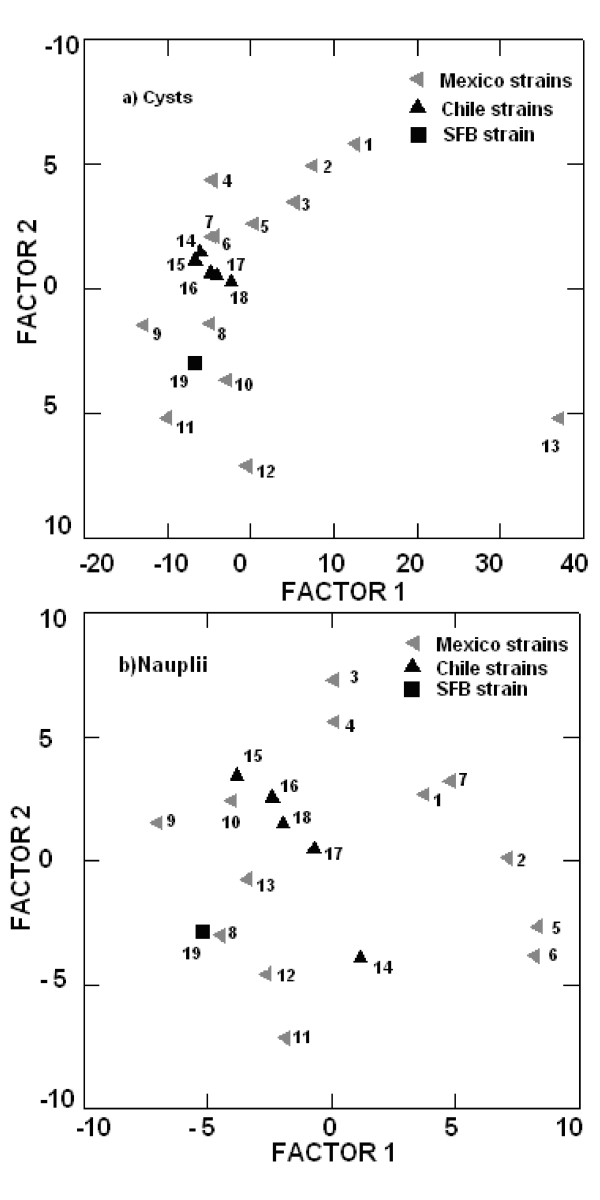
**Discriminant anlyses of *Artemia *cysts (a) and nauplii (b), from México-Chile**. Locations:1 = San Luis Potosí, 2 = Juchitan, 3 = Ohuira, 4 = Yavaros, 5 = Real de las Salinas, 6 = Cuatro Cienegas, 7 = Texcoco, 8 = Zacatecas, 9 = Lobos Bay, 10 = Quintana Roo, 11 = Celestun, 12 = San Crisanto, 13 = El Marquez, 14 = Laguna Cejas, 15 = Poza Pampilla, 16 = Salinas de Cahuil, 17 = salar de Llamara, 18 = Pozas de PlayaYape, 19 = San Francisco Bay.

The discriminant analysis of figure 1 integrates water composition results from both areas. Although variation exists within Mexico and Chile, greater differentiation was observed between habitats at the regional level, i.e., between countries. Surprisingly, a Mexican (Cuatro Ciénegas, *A. franciscana*) and a Chilean location (Torres del Paine, *A. persimilis*) share habitat characteristics, at least for the period when data were collected. Figure 2 shows the discriminat analysis for cyst diameter (a) and nauplius length (b). One location matches cyst diameter with San Francisco Bay (SFB) (Point Lobos) and one (Marquez) is far apart from SFB and all the others. The Chilean locations (Pampilla, Cejar, Cahuil, Llamara, Yape) share cyst diameter, but tend to differ from SFB. The remaining Mexican locations separate well from all the others (Juchitan, Ohuira, Yavaros). Clear differences in cyst diameter are observed among Mexican populations. With regard to nauplii length, populations tend to distribute in a relatively random manner over the two axes, with Marquez, the location greatly differing in cyst diameter to SFB now coming closer to it.

## Discussion

Too often management decisions are taken without the back-up of basic knowledge. Many reasons justify the need to document biometric and ecologic characteristics of *Artemia *sites, either worldwide or at regional level. Firstly, *Artemia *offers a good model to understand how natural populations evolve, considering the isolation and extreme ecological conditions of hypersaline habitats that promote the differentiation of local populations, or adaptations. Hence the speciation mode best describing the *Artemia *situation is adaptive divergence [6,34]. Secondly, local populations exhibit differences in specific phenotypic traits some of which are of practical interest (cyst diameter, nauplii length) for aquaculture. Thirdly, *Artemia *biodiversity is being threatened by the translocation of species and/or populations aimed at improving aquaculture operations or salt production, particularly in developing countries. Last but not least, *A. franciscana *often the best choice for aquaculture, is a successful invader that could threaten locally adapted or native populations or species, as reported in the Western Mediterranean for parthenogenetic types and *A. salina *[12,13]. For these reasons, a biometrical and ecological database should serve as a baseline for further spatial and temporal monitoring of *A. franciscana *and *A. persimilis*. For the former species, relatively pure gene pools would be those located in inland lakes of northern Chile. Additionally, Chile is the southern end for the distribution of *A. franciscana *and normally populations in the edge of distribution tend to differ from those in the center [35]. On the other hand, Mexico is located, relatively, at the central area of distribution for *A. franciscana*, as compared to Chile, and combines the occurrence of exploited and non-exploited salt lakes.

Although this work is aimed to document key biometric and ecological characteristics of *Artemia *sites in both countries, beyond to what has been reported so far [36], this incipient database allows for some initial conclusions. For example, water ionic composition of *Artemia *habitats from Mexico and Chile differ as expected (Fig 1). Some within-country variations exist but it is interesting to see two Chilean (Salar de Atacama; Torres del Paine) and one Mexican population (Cuatro Cienegas) segregated from the rest. This is somewhat correlated with the genetic composition. Salar de Atacama (SAT) is genetically distinct from the rest of the Chilean populations (ascribed to *A. franciscana*), and from San Francisco Bay, the reference sample often used for species verification [37]. Since Torres del Paine (TPA) corresponds to *A. persimilis*, this would be an indication that *A. franciscana *and *A. persimilis *share, to some extent, some habitat characteristics. However, this remains to be proved.

The observed divergence of *Artemia *locations from both areas offers the possibility of finding new populations for aquaculture. As summarized in this paper, only *A. franciscana*, the most widely distributed species in the Americas, is found in Mexico, whilst Chile has both, *A. franciscana *and *A. persimilis*, though separated geographically [30,1], offering an interesting opportunity to understand the ecological separation of these sibling species.

The average cyst diameter reported for *Artemia franciscana *is 237 ± 14 μm, while the average nauplii length is 431 ± 23 μm [3]. As expected, most populations considered in this study are highly heterogeneous, with cyst diameter deviating from the reference sample of *A. franciscana*, except for Lobos in Mexico (see Fig. 2). Although small-sized *A. franciscana *populations (SFB, GSL) are preferred for aquaculture, the heterogeneity in cyst diameter in the samples should be seen as an opportunity for aquaculture diversification, as different species have different larval sizes.

Differential environmental conditions in Mexico and Chile and the magnitude of *Artemia *exploitation in Mexico, which is contrasting to the situation in Chile, are likely to explain the observed north-south cyst differences (cyst tends to be smaller in Chile). Likewise, heterogeneity in cyst diameter in the Mexican samples is greater than in the Chilean ones, which tend to group relatively closer in Fig 2.

Some fundamental and practical conclusions emerge from this study. Habitat heterogeneity, particularly water ionic composition, correlates with heterogeneity observed in cyst diameter and nauplii length. At least for the period considered, Mexican samples showed greater cyst diameter variability, though it is possible that the higher level of human intervention in Mexico plays a role (*Artemia *and salt production often imply population translocation). This might be also a reflection of the higher variability expected at the center of distribution (Mexico) in comparison to populations in the periphery (Chile). Hence, it is clear that biological properties of populations need to be complemented with more careful and systematic description of *Artemia *environments, particularly considering their monitoring on a long-term basis. Updating the database, including other traits, is thus a need for understanding the evolution of *Artemia *populations in nature and the consequences this might have in opening opportunities for invasion or colonization of new populations or species.

## Methods

As this work gathers published information, methodology is extensively described in each of the 21 and 10 papers cited from Mexico and Chile, respectively. Although information is still insufficient, papers from Mexico cover 10 States, whereas that from Chile deals with the two New World species that are found in amazingly contrasting settings: *A. franciscana *in lakes scattered in one of the driest deserts in the world and *A. persimilis *at one of the southernmost latitudes where *Artemia *is found.

Data in the Access file are classified according the following criteria: geographic coordinates, biotope information (pond size, pH, salinity, temperature, cations, e.g., sodium, potassium, calcium, magnesium), and anions (chloride, sulphate, carbonates, and bicarbonates), and biological data (species name, reproductive mode, cyst diameter, chorion thickness. and nauplius length).

### Calculations and statistics

The main ecological characteristics of the different habitats in Mexico and Chile obtained in this paper were analyzed by a multivariate discriminant analysis using Statgraphics (Satistical Graphics Co., Rockville, USA), with the origin of sites taken as the separation factor.

## Authors' contributions

TBC Co-designed the manuscript, participated in Mexican populations data collections and wrote the manuscript.

GG Co-designed the manuscript, participated in Chilean populations data collections and wrote the initial English version of the manuscript.

JMC and GMC participated in Mexican populations data collections, co-edited the manuscript and performed data analyses.

All authors have read and approved the final manuscript.
